# Mechanical, Physical and Thermal Properties of Sugar Palm Nanocellulose Reinforced Thermoplastic Starch (TPS)/Poly (Lactic Acid) (PLA) Blend Bionanocomposites

**DOI:** 10.3390/polym12102216

**Published:** 2020-09-27

**Authors:** A. Nazrin, S. M. Sapuan, M. Y. M. Zuhri

**Affiliations:** 1 Laboratory of Biocomposite Technology, Institute of Tropical Forestry and Forest Products (INTROP), Universiti Putra Malaysia, Serdang 43400, Selangor, Malaysia; nazrinnurariefmardi@gmail.com; 2 Advanced Engineering Materials and Composites Research Centre (AEMC), Department of Mechanical and Manufacturing Engineering, Universiti Putra Malaysia, Serdang 43400, Selangor, Malaysia; zuhri@upm.edu.my

**Keywords:** mechanical properties, poly (lactic acid) (PLA), poly (lactic acid) (PLA) blend bionanocomposites, sugar palm nanocellulose fibre, thermoplastic sugar palm starch

## Abstract

In this paper, sugar palm nanocellulose fibre-reinforced thermoplastic starch (TPS)/poly (lactic acid) (PLA) blend bionanocomposites were prepared using melt blending and compression moulding with different TPS concentrations (20%, 30%, 40%, 60%, and 80%) and constant sugar palm nanocellulose fibres (0.5%). The physical, mechanical, thermal, and water barrier properties were investigated. The SEM images indicated different TPS loading effects with the morphology of the blend bionanocomposites due to their immiscibility. A high content of TPS led to agglomeration, while a lower content resulted in the presence of cracks and voids. The 20% TPS loading reduced the tensile strength from 49.08 to 19.45 MPa and flexural strength from 79.60 to 35.38 MPa. The thermal stability of the blend bionanocomposites was reduced as the TPS loading increased. The thickness swelling, which corresponded to the water absorption, demonstrated an increasing trend with the increased addition of TPS loading.

## 1. Introduction

A huge accumulation of plastic waste has resulted in a negative impact on the environment due to their non-biodegradable properties [1,2]. Plastic products made from synthetics have been used in many industries like the automotive sector, food packaging, furniture, aerospace, defence, electronics, communication, and building construction [3,4,5,6,7,8,9]. These materials have excellent properties: high mechanical strength and stiffness, good chemical and thermal resistances, and impressive barrier properties. However, these petroleum-based polymers have caused massive waste that is detrimental to Mother Nature. Furthermore, the depletion of fossil resources has prompted researchers to develop alternative polymers that are environmentally friendly, which have been derived from biodegradable and sustainable resources [10].

Starch is one of the promising materials that have the potential to replace petroleum-based polymers. It is considered cheap, abundant, biodegradable, and renewable [11,12,13,14,15]. Despite this, starch-based materials are brittle with poor mechanical and water barrier properties. Thus, further modification must be done to acquire more decent material properties. The most common method is by plasticising starch to reduce intermolecular forces and increase the mobility of polymer chains [16,17]. By doing so, plasticised starch or thermoplastic starch (TPS) has a lower glass transition temperature and improved flexibility. Nevertheless, TPS displayed several weaknesses such as poor mechanical strength and water resistance that limit its potential as a packaging material [18]. Reinforcing TPS with natural fibres has been proven to improve the drawbacks of TPS. Natural fibres can be obtained from agricultural crop residues, such as sugar palm, oil palm, pineapple and banana, which amount to billions of tons of waste products around the world [19]. Another approach is to blend natural fibres with different polymers, such as poly (lactic acid) (PLA). PLA is yet another biodegradable polymer that is comparable in terms of its functional properties to synthetic polymers, such as polypropylene (PP), polyethylene (PE), polyethylene terephthalate (PET), and polystyrene (PS) [20]. The downside of PLA is its high production cost, which seems to be non-economical in packaging applications [21]. Hence, starches, natural fibres and plasticisers have often been chosen to reduce the cost of materials and improve their drawbacks.

On this basis, sugar palm starches, like most other starches, have intrigued the interest of fellow researchers in developing starch-based food packaging materials. For poor countries, most starches, such as tapioca, potatoes, wheat, rice, cassava and sago are considered a staple food. The exploitation of such carbohydrates, as matrices in polymer composites, has triggered serious criticism and condemnation. Thus, sugar palm starch is a potential substitution. Sugar palm starch is a high-amylose starch (37%), which can be extracted from sugar palm trees (Arenga pinnata), multipurpose tropical trees that belong to the Palmae family [22]. Starch has limited processability; therefore, plasticisers are required to improve its processability and other properties. The addition of a plasticiser, such as water, glycerol, and sorbitol helped to improve the thermal stability, ductility, cohesion, resistance to retrogradation, and elongation at break of TPS [23]. Aside from their starch, which is extracted from the trunk, almost all parts of the sugar palm tree can be utilised to create significant products. Traditionally, the palm was tapped to obtain the sugar palm sap, which is converted into brown sugar blocks, vinegar, bio-ethanol, and toddy. Sugar palm fibres are naturally woven fibres attached to the trunk and can be used to make sea water-resistant fibres, while the leaves can be used to make brooms, roofing, baskets, matting, and cattle feed [24]. Until recently, sugar palm fibre has been utilised in advanced engineering applications, such as a substitute for glass fibre in the soil stabilisation of road construction, to reinforce polymers, as a matrix composite in material engineering and in underwater cables [25]. Nanocellulose fibres isolated from sugar palm fibres have gained tremendous attraction due to their unique features, such as their excellent mechanical properties, high surface area (100 m^2^ g^−1^), high aspect ratio of 100, light weight, and low density compared to other commercial fibres [26,27,28,29,30,31]. Cao et al. [32] reported that nanocrystalline cellulose has a high theoretical Young’s modulus value of approximately 150 GPa, which is comparable to the value of steel at 200 GPa. The incorporation of nanocellulose fibres into the TPS matrix has reportedly resulted in the high compatibility of 3D hydrogen bonding networks between starch and nanocellulose fibres, thus improving mechanical strength and water barrier properties [33,34]. According to Ilyas et al., sugar palm starch film reinforced with 0.5% sugar palm nanocellulose demonstrated an improvement in both tensile strength (140%) [35] and water vapour permeability (19.94%) [36]. Even so, TPS still does not have the qualities of petrochemical plastics. It was also reported that sugar palm starch and PLA bilayers significantly increased the mechanical resistance and reduced water vapour permeability [37].

Blending TPS and PLA has helped to offset each material’s weakness and potentially overcome the global plastic waste problem. Thus, it is essential to evaluate the performance of TPS/PLA blends as packaging materials that may be beneficial for humans and the ecosystem. To the best of the authors’ knowledge, and from the above literature review, no work has been carried out in the past on the performance of sugar palm nanocellulose fibre-reinforced thermoplastic sugar palm starch/poly (lactic acid) (PLA) blend bionanocomposites. For this reason, the performance of different ratios of TPS/PLA blends mixed in sugar palm nanocellulose fibre composites, which include physical, mechanical, thermal, chemical, and barrier properties, is presented in this paper.

## 2. Materials and methods

### 2.1. Materials

Sugar palm starch was obtained from sugar palm trees located in Jempol, Negeri Sembilan, Malaysia. Matured sugar palm trees were cut and the interior trunk parts were crushed to acquire woody fibres mixed with starch. Water was added to separate the fibres and starch. Since starch is denser than water, the starch sunk into the sedimentation, while the fibre floated to the top. The fibres were removed and the remaining starch was filtered to remove fibrous remnants. The supernatant was discarded, and the wet starch was kept in open air for 48 h, followed by air drying in the oven for 100 °C for 24 h. Poly (lactic acid) (PLA) resin (NatureWorks 2003D) was purchased from Mecha Solve Engineering, Petaling Jaya, Malaysia. Sorbitol and glycerol were used to improve the sugar palm starch’s processability. Table 1 shows the composition of sugar palm starch and various starches.

### 2.2. Sugar Palm Nanocellulose Preparation

Sugar palm fibre can be found entwined naturally from the bottom to the top of the trunk of sugar palm trees. The fibre was cut using an axe and then ground using a Fritsch Pulverisette mill to produce a finer size of 2 mm. The fibre went through a bleaching process to remove lignin-producing holocellulose ASTM D1104-56 [35]. Then, the holocellulose was further treated to obtain α-cellulose according to ASTM D1103-60 (1977) [39]. The acquired cellulose went through acid hydrolysis in which it was soaked in aqueous H_2_SO_4_ (60%) and mechanically stirred at 1200 rpm for 45 min at 45 °C. The ratio of the cellulose to H_2_SO_4_ solution was 5:100 (%). After that, the hydrolysed cellulose was washed four times by centrifugation at 6000 rpm for 20 min at 10 °C. The suspension was dialysed using distilled water to attain a constant pH value (6.5–7.0). The suspension was sonicated for 30 min before being freeze-dried at −110 °C using ethylene gas and then stored prior to sample analysis. The outcomes from the characterization of the sugar palm nanocellulose can be found in work done by Ilyas et al. [35] Table 2 displays the physical properties of sugar palm nanocellulose.

### 2.3. Sample Preparation

**Figure 1:**
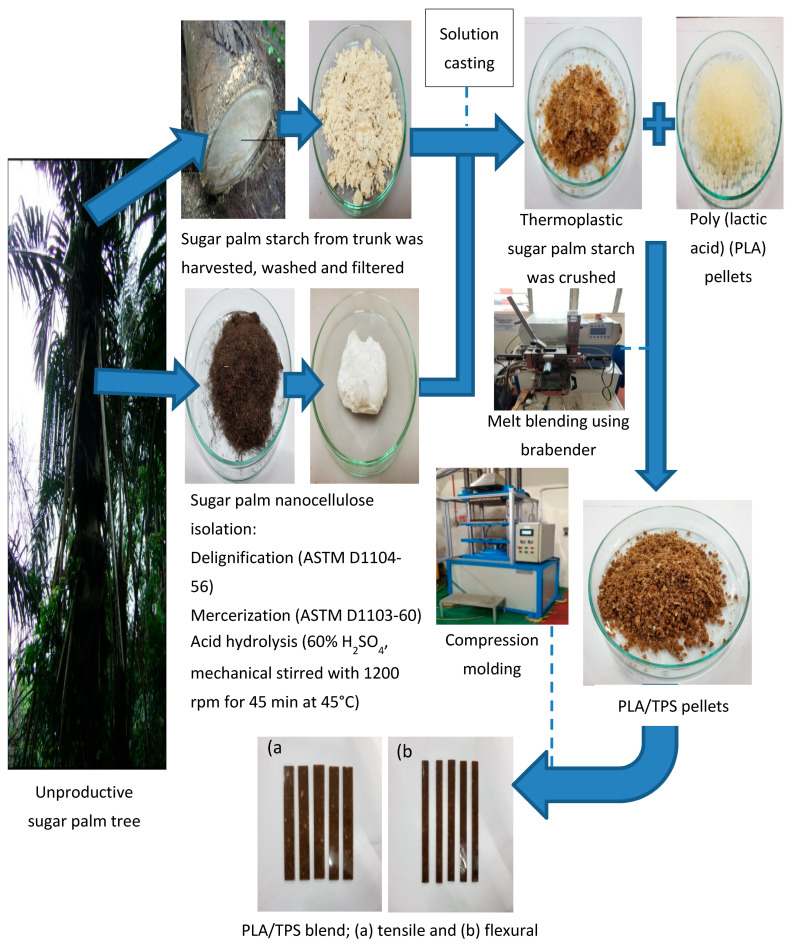
Thermoplastic starch (TPS)/poly (lactic acid) (PLA) blend bionanocomposite preparation.

TPS was prepared using the solution-casting method. Firstly, 100 g of sugar palm starch, 0.5 g of sugar palm nanocrystalline cellulose fibre (0.5%), and 15 g of both glycerol (15%) and sorbitol (15%) were added into 1000 mL of distilled water. The mixture underwent sonication for 15 min to promote sugar palm nanocrystalline cellulose dispersion. The mixture was heated in a water bath at 90 °C for 1 h under continuous stirring. After that, the mixture was poured into glass petri dishes and finally left to dry in the oven at 60 °C for 24 h. PLA and TPS were then melt blended using a Brabender Plastograph (Model 815651, Brabender GmbH & Co. KG, Duisburg, Germany) at 170 °C for 13 min with a rotor speed of 50 rpm. PLA and TPS were mixed into different ratios (80:20, 70:30, 60:40, 40:60, and 20:80). The blend bionanocomposites were crushed into granule size before being hot pressed at 170 °C for 17 min into 150 mm × 150 mm × 3 mm sheets. Figure 1 shows the illustration of preparation of TPS/PLA blend bionanocomposites.

### 2.4. Scanning Electron Microscope (SEM)

The fractured surface of the samples was observed under SEM, model Hitachi S-3400N, operating at an acceleration voltage of 10kV. All samples were coated with gold using an argon plasma metalliser (sputter coater KK575X) (Edwards Limited, Crawley, United Kingdom) to avoid unwanted charging.

### 2.5. Density

The density was determined by using balance (XS205 Mettler Toledo). The sample weight was labelled as m before being submerged into distilled water. The volume of liquid displaced after the submersion was recorded as V and the density ρ was calculated from Equation (1). Five measurements were conducted at 26 °C and the average value was computed.

(1) $$\rho \;=\;\frac{m}{V}$$

### 2.6. FTIR Analysis

Fourier transform infrared (FTIR) spectroscopy was used to detect the presence of functional groups existing in the PLA/TPS blends. The spectra of the material were obtained using an IR spectrometer (Nicolet 6700 AEM). The FTIR spectra of the samples (10 mm × 10 mm × 3 mm) were collected in the range of 4000–400 cm^−1^.

### 2.7. Thermogravimetric Analysis (TGA)

The thermal degradation behaviours of blend bionanocomposites were analysed by thermogravimetric analysis (TGA) with respect to weight loss due to the increase in temperature. TGA was performed with a Q series thermal analysis machine from TA Instruments (New Castle, DE, USA). The analysis was carried out in aluminium pans under a dynamic nitrogen atmosphere in the temperature range 25–900 °C at a heating rate of 10 °C/min.

### 2.8. Water Absorption

Five samples (10 mm × 10 mm × 3 mm) were dried in an air-circulating oven at 100 ± 2 °C for 24 h in order to remove existing moisture and then immersed in water at room temperature (23 ± 1 °C) for 0.5 h and 2 h, as proposed by previous studies. The samples were weighed before (*W*i) and after immersion (*W*f) and the water absorption of the laminates was calculated using Equation (2):

Water absorption (%) = [(*W*f − *W*i)/*W*i] × 100 (2)

### 2.9. Thickness Swelling

Five samples (10 mm × 10 mm × 3 mm) were dried in an air-circulating oven at 100 ± 2 °C for 24 h in order to remove existing moisture and then immersed in water at room temperature (23 ± 1 °C) for 0.5 h and 2 h, as proposed by previous studies. The thickness swelling of samples was measured before (*T*i) and after immersion (*T*f) using a digital Vernier (Model: Mitutoyo) with 0.01-cm accuracy. The thickness swelling ratio of the blend was calculated using Equation (3):

Thickness swelling (%) = [(*T*f − *T*i)/*T*i] × 100 (3)

### 2.10. Tensile Testing

Tensile tests were conducted according to ASTM D-638 at a temperature of 23 ± 1 °C and a relative humidity of 50 ± 5%. The tests were carried out on five repetitions using a Universal Testing Machine (INSTRON 5556) with a 5-kN load cell; the crosshead speed was maintained at 1 mm/min.

### 2.11. Flexural Testing

Flexural tests were conducted according to ASTM D-790 at a temperature of 23 ± 1 °C and a relative humidity of 50 ± 5%. The samples were prepared with dimensions of 130 mm (*L*) × 13 mm (*W*) × 3 mm (*T*). The tests were carried out with five repetitions using a Universal Testing Machine (INSTRON 5556) with a 5-kN load cell; the crosshead speed was maintained at 1 mm/min. The support span length was set at a ratio of 16:1 to the thickness of the samples.

## 3. Results and Discussion

The results and a discussion of the performance of the nanocellulose fibre-reinforced PLA/TPS blend bionanocomposites are presented in the following subsections.

### 3.1. FTIR Analysis

**Figure 2:**
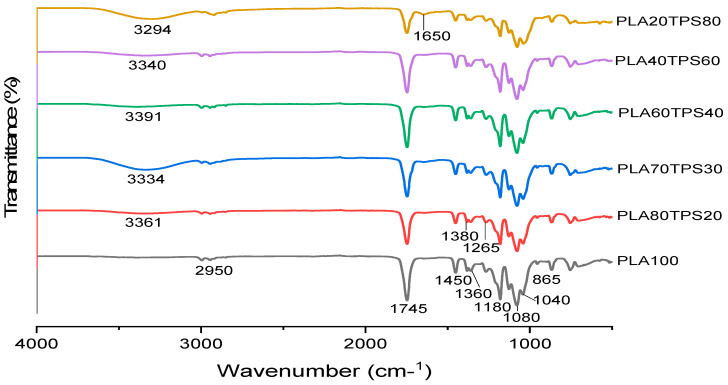
FTIR spectra of PLA100 and different ratios of PLA/TPS blend bionanocomposites.

The FTIR data were analysed to demonstrate the possible physical and chemical interactions between PLA, TPS, and nanocellulose fibres. Figure 2 shows the FTIR spectrum curves of the PLA100 and PLA/TPS blend bionanocomposites of different ratios. It was found that the FTIR spectrum for PLA100 and PLA/TPS blend bionanocomposites showed quite similar peaks. With the exception of PLA100, all blend bionanocomposite spectra showed broad absorption bands ranging from 3200 to 3500 cm^−1^, which indicated the presence of O–H groups in starch, glycerol, and cellulose fibre associated with free, inter-, and intramolecular bound hydroxyl groups. However, PLA70TPS30 and PLA20TPS80 showed a higher wavenumber shift in the O–H band compared to other bionanocomposites. The shifted wavenumber implied weak inter and intramolecular hydrogen bonds between starch chains. These results reflect that blending PLA with starch hindered the hydrogen bonds of starch molecules. The IR spectra of PLA100 and all blend bionanocomposites for C–H stretching can be observed at 3000–2875 cm^−1^. The sharp absorption peak at approximately 1745 cm^−1^ might be associated with the –C=O stretching vibration of the ester group in PLA. With the addition of TPS, the peak seems to shift to a lower wavenumber, which can be seen in all bionanocomposite samples. The function groups might be affected by the weak interaction between these two immiscible polymers. According to Wan et al. [40], the carbonyl groups also played a role in the interaction between PLA and TPS, increasing the dispersion of the two phases. The peaks at approximately 1450 and 1360 cm^−1^ were assigned to asymmetric and symmetric –CH_3_ deformation vibrations, respectively [41]. The sharp absorption peaks at 1180, 1080, and 1040 cm^−1^ represent the C–O stretching vibration [42]. After TPS addition, these peaks can be seen to have shifted to a lower wavenumber. These phenomena are similar to the shifted wavenumber at 1745 cm^−1^. At the 865 cm^−1^ absorption peak, the presence of C–C bonding was detected. Figure 2 shows that all blend bionanocomposites have predominant functional groups of hydroxyl, whereas PLA100 has the highest carbonyl groups.

### 3.2. Morphological Analysis

**Figure 3:**
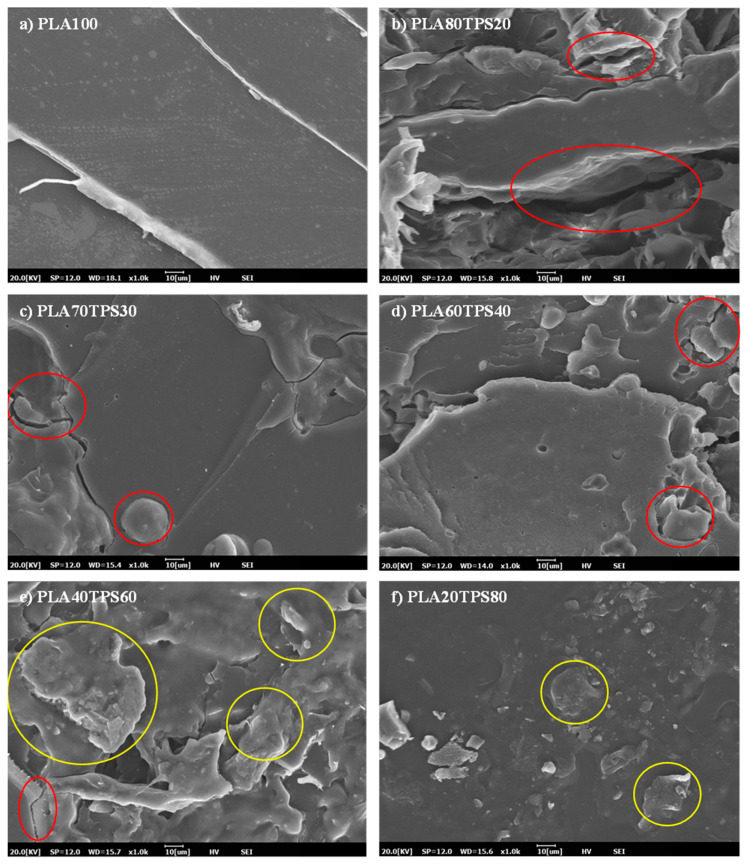
SEM images of tensile fractured surfaces for (**a**) PLA100, (**b**) PLA80TPS20, (**c**) PLA70TPS30, (**d**) PLA60TPS40, (**e**) PLA40TPS60, (**f**) PLA20TPS80.

Figure 3 shows the SEM images of the tensile fractured surfaces of PLA100 and the PLA/TPS blend bionanocomposites. In general, the SEM images showed that good dispersion was achieved by using the combination of glycerol, sorbitol, and water as plasticisers. As reported by Wang et al. [43], the substitution of a 10% weight of glycerol with water resulted in no visible starch granules on the fracture surface of PLA/TPS blend bionanocomposites. However, Li and Huneault [44] performed a study on the comparison of glycerol and sorbitol on a PLA/TPS blend and found out that the substitution of glycerol with sorbitol decreased the particle size and promoted a fine dispersion of TPS. As for Figure 3b, the blend bionanocomposites showed a dispersion network with a co-continuous structure with flakes and less defined edges. This co-continuous morphology was usually obtained after mixing immiscible polymer pairs. The structure was formed by the melt mixing of polymers, allowing different structure types to coexist rather than forming a homogeneous network [45]. Figure 3c demonstrates a similar smooth fracture surface to PLA100 but with some crack-spreading areas (red circles). Yokesahachart and Yoksan [46] performed a similar study of PLA70TPS30 and the morphology of the blends was identical to that of neat PLA with a smooth crack propagation area. Meanwhile, Figure 3d demonstrates the voids and ragged fracture surface of the blend bionanocomposites, which lead to brittle fractures. Wang et al. [43] obtained quite similar results where the PLA/TPS blend displayed a rugged surface and deteriorated dispersion between PLA and TPS, which suggested some incompatibility between PLA and TPS. As the TPS content is increased Figure 3e, the cracks become smaller and only small voids are visible, but clear agglomerations can be seen (yellow circles), suggesting the presence of undisrupted starch granules within the PLA matrix [21]. Figure 3f shows smooth fracture surfaces that are quite similar to PLA100 with lesser agglomerations, suggesting the brittleness and rigidity of the blend bionanocomposites [46].

### 3.3. Density

**Figure 4:**
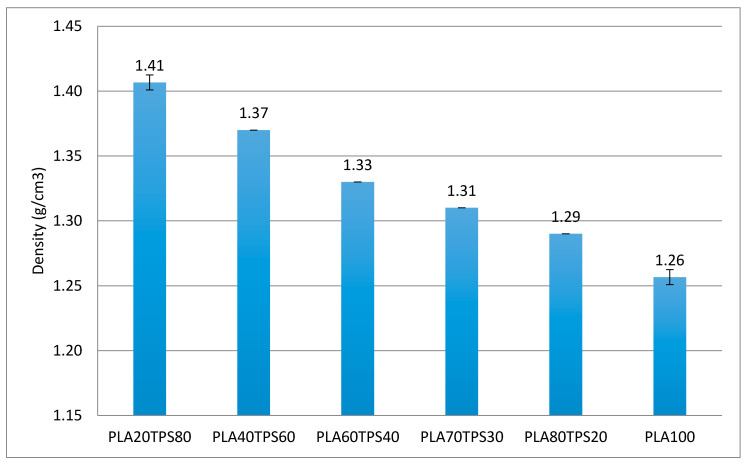
Density of PLA100 and PLA/TPS blend bionanocomposites.

Based on Figure 4, it is observed that PLA100 has the lowest density (1.26 g/cm^3^) followed by the blend bionanocomposites with the lowest TPS content (1.29 g/cm^3^). As suspected, increasing the TPS content resulted in increased density in the blend bionanocomposites as well. Similar results were reported by Sanyang et al. [37], where the density of sugar palm starch–PLA bilayer films decreased as the proportion of PLA was reduced. Density is closely related to molecular weight. A high-density material is associated with a high molecular weight. Amylose has a molecular weight around 80000–1000000 g/mol, which is 10 times higher than conventional synthetic polymers, such as PE, PP and PS, while amylopectin has a molecular weight much greater than amylose [47]. In this case, starch is well known for its brittleness, which can be assumed to be the reason for the mechanical properties’ deterioration in the PLA/TPS blend bionanocomposites. However, there was no major influence of the incorporation of nanocrystalline cellulose (NCC) on the changes in density of the blend bionanocomposites. Ilyas et al. [35] reported that there was no significant change in density with low concentrations of the nanofiller sugar palm nanocrystalline cellulose.

### 3.4. Mechanical Properties

**Figure 5:**
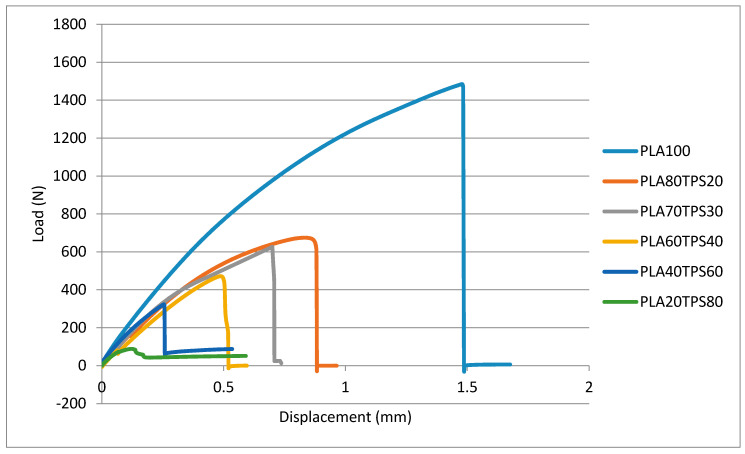
Tensile load–displacement curves of PLA100 and PLA/TPS blend bionanocomposites.

**Figure 6:**
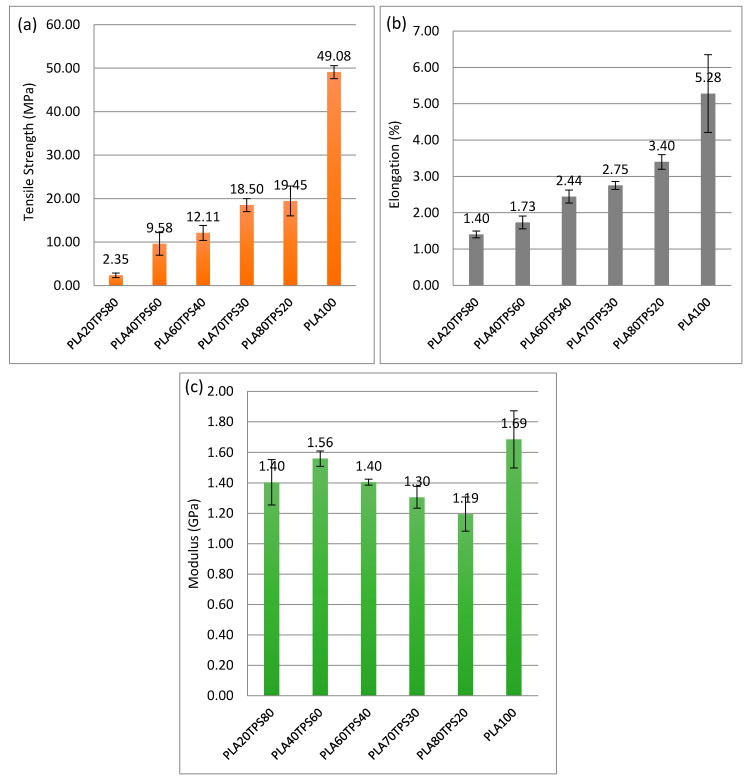
(**a**) Tensile strength, (**b**) elongation at break, (**c**) Young’s modulus of PLA100 and PLA/TPS blend bionanocomposites.

In terms of mechanical properties, PLA demonstrates excellent polymer properties comparable to synthetic polymers. However, the high price tag due to its complex production process is not economical for food packaging applications. As a step to reduce the cost, TPS was chosen to be blended with PLA, producing an acceptable standard of mechanical properties for packaging materials. There were five ratios of PLA/TPS blend bionanocomposites with different TPS contents: 20%, 30%, 40%, 60% and 80% (*w/w*). Figure 5 gives the tensile load–displacement curves for PLA100 and PLA/TPS blend bionanocomposites. In the figure, the addition of 20% of TPS content decreased the tensile load (0.67 kN) before fracture, exceeding 50% compared to PLA100 (1.46 kN). However, further addition up to 30% only decreased the tensile load to 0.63 kN. Both tensile load and displacement values kept on decreasing as TPS content increased. Referring to Figure 6, as expected, PLA100 or neat PLA possessed superior mechanical properties, surpassing all the blend bionanocomposites. The addition of TPS into PLA decreased 60% of the tensile strength from 49.08 to 19.45 MPa with just 20% of TPS addition. It is well known that PLA and TPS are chemically incompatible and TPS might have contributed to the brittleness of the PLA blend [48]. The same goes for the elongation at break, where PLA100 had the highest value (5.28%) and the values kept on decreasing as TPS was added. Similar results were obtained by Wokadala et al. [49], where every 10% addition of waxy starch decreased the elongation at break. There was only a slight difference in the tensile strength values of PLA80TPS20 and PLA70TPS30, which were 19.45 and 18.50 MPa, respectively. Akrami et al. [50] fabricated a PLA/TPS blend (70:30) using melt blending and compression moulding, which produced quite similar values of tensile strength (18 MPa) but lower values of elongation at break (1.34%). This may be due to the incompatibility of the PLA matrix with the dispersed phase. The tensile strength value of PLA60TPS40 (12.11MPa) dropped drastically compared to PLA70TPS30 (18.5MPa), which was about 34.5%. This can be related to the SEM imaging of PLA60TPS40 with some voids and a ragged structure that caused brittle fractures. As can be seen from the morphology of the polymer blend bionanocomposites with higher TPS contents (PLA40TPS60 and PLA20TPS80), partial agglomeration can be found, suggesting poor interfacial adhesion between PLA and TPS. This resulted in a further decrease in the tensile strength [51]. This verified that every increment of TPS content into PLA led to a decline in tensile strength and further elongation at break due to the brittleness of TPS. Even so, the increasing addition of TPS appeared to improve the Young’s modulus.

**Figure 7:**
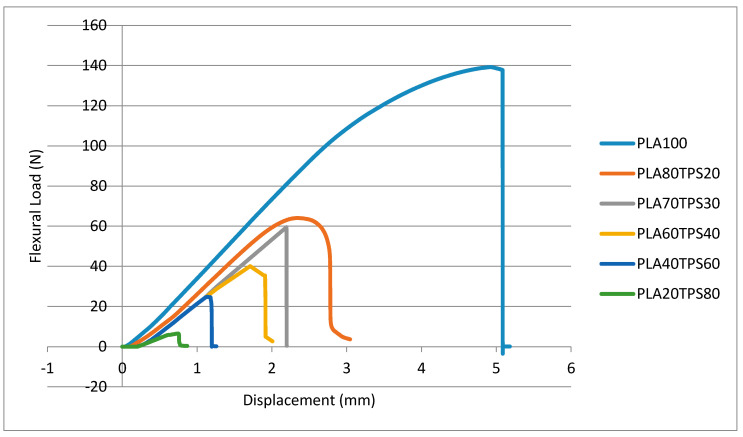
Flexural load–displacement curves of PLA100 and PLA/TPS blend bionanocomposites.

**Figure 8:**
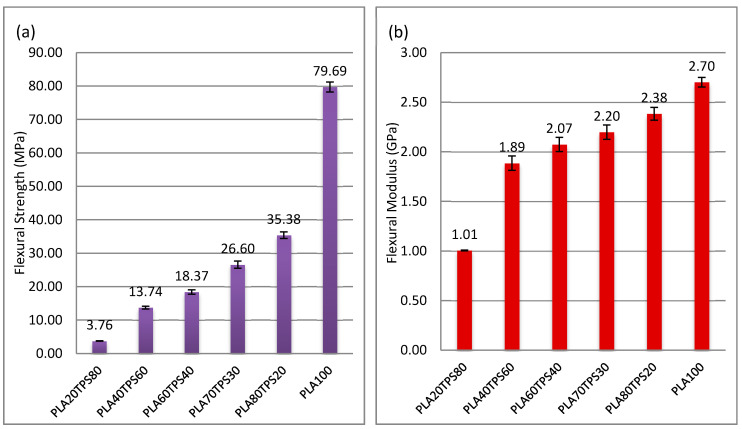
(**a**) Flexural strength, (**b**) flexural modulus of PLA100 and PLA/TPS blend bionanocomposites.

Figure 7 demonstrates flexural load–displacement curves that are quite identical to tensile load–displacement curves. Introducing 20% of TPS causes a huge drop in flexural load value before fracture. There is only a slight change in the flexural load values of PLA80TPS20 (0.064 kN) and PLA70TPS30 (0.0059 kN). For both tensile load–displacement and flexural load–displacement curves, the displacement before fracture decreased as the TPS content increased. The brittleness of TPS is responsible for these drops. Figure 8a,b show the flexural strength and modulus of PLA100 and PLA/TPS blend bionanocomposites, respectively. As observed, a quite similar trend to tensile strength and elongation at break was obtained. The incorporation of TPS into PLA seemed to reduce the flexural properties of the blend bionanocomposites. Adding 20% of TPS sharply decreased the flexural strength from 79.60 to 35.38 MPa, which equates to 55%. The flexural strength value of PLA80TPS20 compared to PLA70TPS30 seemed to decrease by about 25%, which equates to 26.60 MPa. According to Raghu et al. [21], the flexural strength and modulus of PLA70TPS30 was obtained using injection moulding, and were 24.12 MPa and 2.4 GPa, respectively, nearly the same as the results of the materials in this study. The same blend of bionanocomposites with the incorporation of wood fibres (20%) significantly improved the flexural strength and modulus to 30.25 MPa and 2.6 Gpa, respectively. As the addition of TPS loading increased, the flexural properties of the blend bionanocomposites decreased, similar to the tensile properties. Likewise, Ferri et al. [23] revealed blend bionanocomposites with increments of 5% TPS, whereby PLA acquired similar decreasing trends of flexural strength and modulus. The plasticiser combination of glycerol and sorbitol improved the mechanical properties of the blend bionanocomposites. The incorporation of sorbitol promoted a finer and more uniform distribution of TPS within the PLA. Meanwhile, glycerol contributed to the better elongation at break [52]. A good ratio between PLA and TPS improved the dispersion of TPS into the PLA matrix, which resulted in better tensile strength.

### 3.5. Thermal Properties

**Figure 9:**
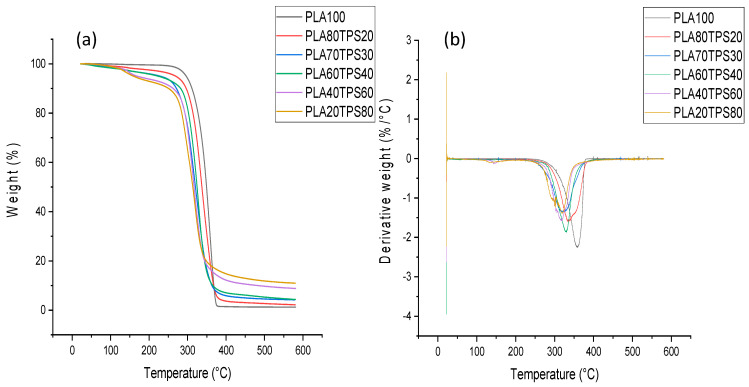
(a) Thermogravimetric analysis (TGA), (b) derivative thermogravimetric (DTG) curves of PLA100 and PLA/TPS blend bionanocomposites.

Thermogravimetric analysis (TGA) and derivative thermogravimetric (DTG) curves were employed to investigate the thermal stability and decomposition of polymeric systems. The prominent peak in the DTG curves and the specific weight loss in the TGA graph are associated with each thermal degradation stage. It is important to acquire the statistics regarding degradation and decomposition mode under heat exposure in order to optimise the processing capabilities of a polymer [53]. Figure 9a,b show the results of the TGA and DTG curves of PLA100 and PLA/TPS blend bionanocomposites, which demonstrated three thermal degradation stages where the initial stage happened at a temperature below 100 °C. According to Sanyang et al. [22], the mass loss within this stage can be related to the evaporation or dehydration of poorly bound water and low molecular weight compounds in the blend bionanocomposites. Table 3 shows the thermal degradation temperatures at 5% (*T*_5%_), 25% (*T*_25%_), 50% (*T*_50%_) and 75% (*T*_75%_) mass losses and maximum weight loss (*T*_max%_). At 5% weight loss, the degradation temperature of bionanocomposite samples drop as TPS content is added. However, PLA80TPS20 and PLA70TPS30 (222 °C) demonstrate a similar degradation temperature in the same way as PLA60TPS40 and PLA40TPS60 (158 °C). There is a huge drop in the degradation temperature from PLA60TPS40 to PLA40TPS60. This change is related to the higher concentration of glycerol in the higher proportion of TPS. The volatilisation of hydrolysed glycerol occurs at 160–195 °C. The maximum weight loss (*T*_max%_) for all blend bionanocomposites observed in the temperature range between 343–376 °C, indicating the maximum degradation of PLA and TPS. The char residue significantly increased as the TPS content increased since starch produces a higher yield of charred products compared to PLA.

The second stage of the thermal degradation occurred within the varying range for all blend bionanocomposite samples. As observed in Figure 9, the increasing addition of TPS resulted in a greater mass loss. PLA80TPS20 had the highest temperature range of 130–280 °C. A similar degradation temperature range was observed for PLA70TPS30 and PLA60TPS, which was 130–265 °C. The thermal degradation temperature dropped to 130–250 °C for both of the lowest PLA content samples (PLA40TPS60 and PLA20TPS80). Glycerol has a boiling point of 290 °C, but, in this case, starch with a helical structure of all hydroxyl groups formed hydrogen bonds with glycerol and subsequently lowered the volatility of glycerol in the blend bionanocomposite samples [54]. This stage was associated with the volatilisation of plasticiser compounds by various authors [18,21,22,35,55].

The drastic weight reduction that occurred for all samples indicated the highest thermal degradation with the temperature beyond 250 °C. At this stage, all samples were attributed to polymer matrix decomposition. PLA100 had the highest thermal stability; thus, further addition of TPS content in the blend bionanocomposites lowered the thermal stability [40]. The maximum degradation rate displayed a varying temperature with PLA100 having the highest value (376 °C) followed by PLA80TPS20 (373 °C), PLA70TPS30 and PLA60TPS40 (361 °C), and PLA40TPS60 and PLA20TPS80 (343 °C). These shifting temperatures are associated with the decomposition of starch at 280–320 °C and the decomposition of PLA at 330–360 °C [56].

### 3.6. Water Absorption

It is well known that starch-based materials are sensitive to water which is why it is crucial to investigate their sensitivity when blended with hydrophobic polymers for efficient packaging applications. The water absorption of PLA100 and PLA/TPS blend bionanocomposites after 0.5 and 2 h of immersion is displayed in Table 4. Obviously, the longer period of immersion resulted in a higher water absorption for all blend bionanocomposite samples. Moreover, a higher concentration of TPS led to even quicker water absorption. At the 0.5 h immersion period, increasing the TPS content by 10% showed a drastic increment in water absorption from PLA80TPS20 (0.93%) to PLA70TPS30 (6.23%) and PLA60TPS40 (11.18%). This can be associated with the presence of cracks and voids as mentioned in the morphological analysis due to the lack of interfacial bonding between the starch and PLA matrix [46]. As for PLA40TPS60 (17%) and PLA20TPS80 (22.4%), the water absorption values increased, respectively, with 20% of TPS variation. The water absorption of all blend bionanocomposite samples skyrocketed after the 2 h immersion period.

### 3.7. Thickness Swelling

The swelling behaviour was studied to investigate the dimensional stability of the materials for packaging application purposes. Thickness swelling is often associated with the moisture absorption capability of the material [57]. The thickness swelling results of PLA100 and PLA/TPS blend bionanocomposites for 0.5 h and 2 h of immersion can be observed in Table 4. Based on the results, all bionanocomposites samples followed a similar trend as the water absorption while PLA100 remaining undisturbed, The bionanocomposites samples showed an increasing trend as TPS loading was increased. For the 0.5-h immersion period, a major increment in swelling values can be seen from PLA80TPS20 (0.9%) to PLA70TPS30 (2.35%) and PLA60TPS40 (10.72%). After that, the addition of 20% TPS content resulted in a further increase in swelling values for PLA40TPS60 (11.41%) and PLA20TPS80 (16.08%). As for 2 h of immersion, it was observed that every blend bionanocomposite sample went through a drastic increase in swelling value. All blend bionanocomposite samples showed a beyond 100% swelling rate compared to 0.5 h of immersion. Teixeira et al. [58] found out that the water absorption of cassava bagasse nanofibrillated cellulose reinforced thermoplastic cassava starch was reduced by 32–37% with the incorporation of only glycerol and by 15–18% with both glycerol and sorbitol.

## 4. Conclusions

From this study, the effect of different ratios of TPS/PLA blends on sugar palm nanocellulose fibres helped to reinforce thermoplastic sugar palm starch/poly (lactic acid) (PLA) blend bionanocomposites, as their physical, mechanical and thermal properties were evaluated. A series of PLA/TPS blend bionanocomposites were produced using melt blending and compression moulding with varied loadings of TPS (20%, 30%, 40%, 60% and 80%) and at a constant loading of sugar palm nanocellulose fibres (0.5%). Referring to Table 5, the experimental results demonstrated that the increase in the TPS loading decreased the overall mechanical properties of the blend bionanocomposites due to the brittleness of TPS. In addition, there was an increase in the Young’s modulus up to 60% of TPS loading from 1.19 to 1.56 GPa. All bionanocomposite samples except PLA20TPS80 possess acceptable values of mechanical properties for packaging applications, considering no compatibiliser was used. As for FTIR analysis, PLA70TPS30 and PLA20TPS80 displayed a higher shifted wavenumber of O–H bands compared to other bionanocomposites. These results can be related to the apparent increase in the water absorption of these two samples from the previous samples. The SEM micrograph images of the fracture surfaces showed voids and crack- spreading areas for blend bionanocomposites with TPS loadings under 50%. Meanwhile, some agglomerations were visible for blend bionanocomposites with TPS loadings beyond 50%. This result can be related to the incompatibility of the starch and PLA matrix without the incorporation of a compatibiliser to enhance its miscibility. The thermal stability decreased as the TPS loading increased from 373 to 343 °C. In Table 3, it is observed that PLA60TPS40 demonstrated a higher degradation temperature at 25% (307 °C) and 50% (324 °C) weight losses compared to PLA70TPS30. It is assumed that PLA60TPS40 has a better affinity between PLA and TPS. The density, water absorption, and thickness swelling increased as the TPS loading increased, which can be associated with the predominant functional group of hydroxyls. However, there was no significant effect of the sugar palm nanocellulose fibres on the blend bionanocomposites due to a minimal amount being added. In a nutshell, PLA60TPS40 can be considered to achieve reasonable functional properties for food packaging applications. Hence, this study reveals the potential of PLA/TPS blend bionanocomposites for biodegradable packaging applications.

## Figures and Tables

**Table 1:** The composition of sugar palm starch and various starches [38].

Starch	Density	Water Content (%)	Amylose (%)
Tapioca	1.446–1.461	13	17
Sago	-	10–20	24–27
Potato	1.54–1.55	18–19	20–25
Wheat	1.44	13	26–27
Maize	1.5	12–13	26–28
Sugar palm	1.54	15	37.60

**Table 2:** Physical properties of sugar palm nanocellulose.

Properties	Value
Diameter (nm)	9
Density (g/cm^−3^)	1.05
Moisture content (wt %)	17.90
Degree of crystallinity (%)	85.9
Surface area (m^2^/g)	14.47
Pore volume (cm^3^/g)	0.226
Degree of polymerization	142.86
Molecular weight (g/mol)	23164.7

**Table 3:** Thermal degradation temperature of PLA100 and PLA/TPS blend bionanocomposites.

Sample	*T*_5%_ (°C)	*T*_25%_ (°C)	*T*_50%_ (°C)	*T*_75%_ (°C)	*T*_max_ (°C)	Char Residue (%)
PLA20/TPS80	158	290	313	335	343	10.969
PLA40/TPS20	158	298	313	335	343	8.824
PLA60/TPS40	222	307	324	339	361	4.372
PLA70/TPS30	222	298	320	339	361	4.232
PLA80/TPS20	286	319	337	354	373	2.198
PLA100	296	332	349	360	376	1.247

**Table 4:** Density, thickness swelling and water absorption of PLA100 and PLA/TPS blend bionanocomposites.

Sample	Density (g/cm^3^)	Thickness Swelling (%)		Water Absorption (%)	
		0.5 h	2 h	0.5 h	2 h
PLA20/TPS80	1.41 ± 0.01	16.08 ± 1.8	40.17 ± 1.58	22.40 ± 0.95	61.24 ± 0.85
PLA40/TPS60	1.37 ± 0.01	11.41 ± 0.3	31.66 ± 1.61	17.0 ± 0.93	46.42 ± 0.84
PLA60/TPS40	1.33 ± 0.01	10.72 ± 0.86	25.97 ± 2.02	11.18 ± 0.62	25.21 ± 0.37
PLA70/TPS30	1.31 ± 0.01	2.35 ± 0.57	11.23 ± 0.92	6.23 ± 0.27	14.49 ± 0.51
PLA80/TPS20	1.29 ± 0.01	0.90 ± 0.21	4.18 ± 0.82	0.93 ± 0.28	5.70 ± 0.37
PLA100	1.26 ± 0.01	0	0	0	0

**Table 5:** Mechanical properties of PLA100 and PLA/TPS blend bionanocomposites.

Sample	Tensile Strength (MPa)	Young’s Modulus (GPa)	Elongation (%)	Flexural Strength (MPa)	Flexural Modulus (GPa)
PLA20/TPS80	2.35	1.40	1.40	3.76	1.00
PLA40/TPS60	9.58	1.55	1.73	13.74	1.88
PLA60/TPS40	12.11	1.40	2.44	18.37	2.07
PLA70/TPS30	18.50	1.30	2.75	26.60	2.19
PLA80/TPS20	19.45	1.19	3.40	35.38	2.38
PLA100	49.08	1.68	5.28	79.69	2.70
